# Mangrove health assessment using spatial metrics and multi-temporal remote sensing data

**DOI:** 10.1371/journal.pone.0275928

**Published:** 2022-12-06

**Authors:** Pham Minh Hai, Pham Hong Tinh, Nguyen Phi Son, Tran Van Thuy, Nguyen Thi Hong Hanh, Sahadev Sharma, Do Thi Hoai, Vu Cong Duy

**Affiliations:** 1 Vietnam Institute of Geodesy and Cartography, Ministry of Natural Resources and Environment, Hanoi, Vietnam; 2 Faculty of Environment, Hanoi University of Natural Resources and Environment, Hanoi, Vietnam; 3 Faculty of Environmental Sciences, VNU University of Science, Vietnam National University, Hanoi, Vietnam; 4 Institute of Ocean and Earth Sciences, University of Malaya, Kuala Lumpur, Malaysia; 5 Faculty of Information Technology, Hanoi University of Science and Technology, Hanoi, Vietnam; Đại Học Duy Tân: Dai Hoc Duy Tan, VIET NAM

## Abstract

Mangrove forest plays a very important role for both ecosystem services and biodiversity conservation. In Vietnam, mangrove is mainly distributed in the Mekong delta. Recently, mangrove areas in this region decreased rapidly in both quality and quantity. The forest became bare, divided and scattered into many small patches, which was a major driver of ecosystem degradation. Without a quantitative method for effectively assessing mangrove health in the regional scale, the sustainably conserving mangrove is the challenge for the local governments. Remote sensing data has been widely used for monitoring mangrove distributions, while the characterization of spatial metrics is important to understand the underlying processes of mangrove change. The objectives of this study were to develop an approach to monitor mangrove health in Mui Ca Mau, Ca Mau province of Vietnam by utilizing satellite image textures to assess the mangrove patterns. The research result showed that mangrove areas increased double by 2015, but the forest had become more fragmented. We can be seen those changes in land use mainly come from land conversion from forest to shrimp farms, settlements areas and public constructions. The conserving existing mangrove forest in Mui Ca Mau should consider the relations between mangrove health and influencing factors indicated in the manuscript.

## Introduction

Mangrove forests are among the earth’s most diverse and dynamic ecosystems [1]. They offer various ecosystem services and economic values in stabilizing shorelines, coastal habitat and biodiversity protection for marine and pelagic species, coastal protection from storm surges, fisheries and forestry products such as fuel, medicine, and food for local communities [2]. Despite their importance, mangrove forests have been rapidly disappearing at an alarming rate across the globe—at least 23% of mangrove forests have been lost in the past two decades [3]. Forest loss often associated with a loss in terms of degradation, which theoretically reflects the balance of biodiversity and ecosystem’s function.

The remote sensing is an advanced technique due to its synoptic and repeated coverage, low-cost or free of charge, and availability of historic satellite data [4]. Remote sensing data can (i) provide historical and current information about the status of mangrove in large areas; (ii) produce high spatial, spectral and temporal resolutions for quantitative and qualitative measurements. Many studies conducted on mangrove ecosystems have used multi-spectral satellite data for determining spatio-temporal change [5–15]. The remote sensing has also been widely used to monitor mangrove distribution [16–20]. In particular, the current trend is to use patterns on remote sensing images to monitor the structure of mangroves based on their distributions [6, 21].

In order to support remote sensing classifications in analyzing mangrove change patterns overtime, spatial metrics can be used. Spatial metrics are defined as landscape indicators to describe the morphology and structure of a landscape [22, 23]. Spatial metrics are measurements derived from digital analysis of thematic categorical maps exhibiting spatial heterogeneity at a specific scale and resolution [24]. Consuration of a landscape refers to the spatial arrangement, position, orientation and shape complexity of patches in the landscape. There has been considerable interest recently in using spatial information-derived images and landscape metrics to describe mangrove forest change. Spatial metrics have been applied to investigate the connectivity, fragmentation, configuration, and complexity of ecosystems [24, 25]. Spatial metric can be estimated at three levels: patch, class, and landscape [26, 27].

Many studies have been conducted on the mangrove forest in Vietnam using remote sensing techniques. Most of them have concentrated on monitoring land use changes and mapping forest areas, but measuring mangrove health is not comprehensive. There is growing concern over the conservation and management of mangrove in Vietnam, which has spurred local managers to seek a better approach to answer the questions of where mangrove forest degradation and how to access mangrove health at a variety of spatial and temporal scales. The combination of remote sensing technology and spatial metrics, in particular, has made a powerful approach for analyzing and managing mangrove degradation based on their distribution structures [28], which focused to quantify 3 characteristics of the landscape [29] as: *Structure*, the spatial relationships among the distinctive ecosystems or “elements” present—more specifically, the distribution of energy, materials, and species in relation to the sizes, shapes, numbers, kinds, and configurations of the ecosystems; *Function*, the interactions among the spatial elements, that is, the flows of energy, materials, and species among the component ecosystems; *Change*, the alteration in the structure and function of the ecological mosaic over time. Therefore, the specific objectives of this study were: 1) To determining factors influencing mangrove health; 2) To characterize the mangrove health in Mui Ca Mau, Ca Mau province of Vietnamby using remote sensing and spatial metrics.

## Study area and data sets used in the study

### Study area

**Fig 1:**
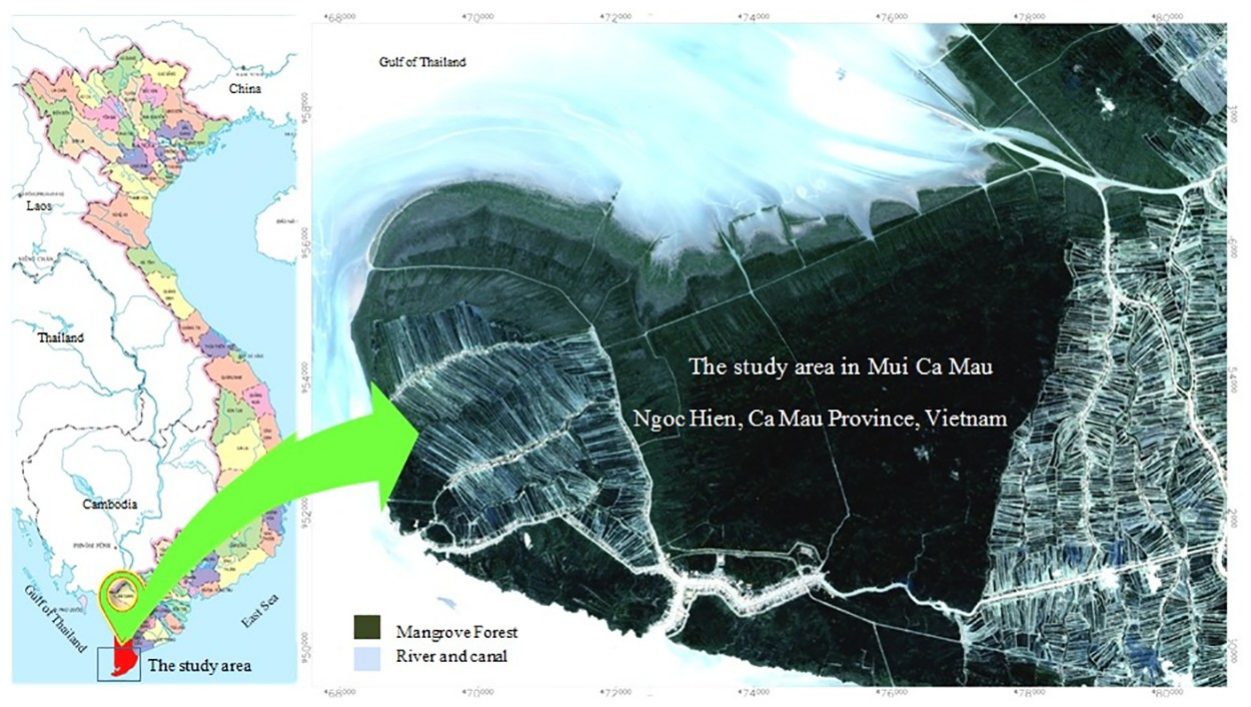
Study area (republished satellite data under a CC BY license, with permission from the Department of Remote Sensing, Ministry of Natural Resources and Environment of Vietnam, original copyright [1995, 2004, 2015]).

The study area was Mui Ca Mau located on Ca Mau Peninsula in Dat Mui commune, Ngoc Hien district, Ca Mau province of Vietnam (Fig 1). The total area is 41,862 ha, of those inland territory is 15,262 ha, and mudflat and tidal area is 26,600 ha. Mui Ca Mau has a low terrain with tropical climate and monsoon season, the rainy season from May to November and dry season from December to April. The average air temperature recorded is 27°C. The rainfall was around 2200 mm with highest in October recorded at 500 mm [30]. The area boasts beautiful land and seascapes and a high biodiversity of marine areas and swamp wetlands. There are three types of ecosystems: marine, mangrove and a dense tropical rainforest ecosystem. The representative mangroves forest area: *Rhizophora apiculata*, *Avicennia alba*, Mixed *R*. *apiculata—A*. *alba* (*R*. *apiculata* dominated), Mixed *A*. *alba—R*. *apiculata* (*A*. *alba* dominated). The mangroves in Mui Ca Mau have been qualitatively and quantitatively degrading due to various anthropogenic causes like the over-exploitation of natural resources, pollution from rural and urbanized areas, especially the extensive development of shrimp farms [30–32].

### Data sets used in the study

In this research, SPOT satellite images acquired in 1995 (SPOT4), 2004 (SPOT5), 2015 (SPOT5), provided by the Department of Remote Sensing, Ministry of Natural Resources and Environment of Vietnam, were used due to their ability to provide high ground resolution, and the red and NIR spectral bands for suitable vegetation indices [33]. All satellite data were acquired during the summer months from November to December to reduce the effects of seasonal change on land cover. Each scene had a cloud cover of less than 10%. Satellite images then adjusted to a standard Universal Transverse Mercator (UTM) coordinate system. To conduct a quantitative comparison of the satellite images, the fusion of panchromatic and multispectral satellite images was processed and resampled to the spatial resolution of SPOT 6 (1.5 m). The resampling process was conducted using the Registration tool in the image-processing package Envi 4.3.

**Fig 2:**
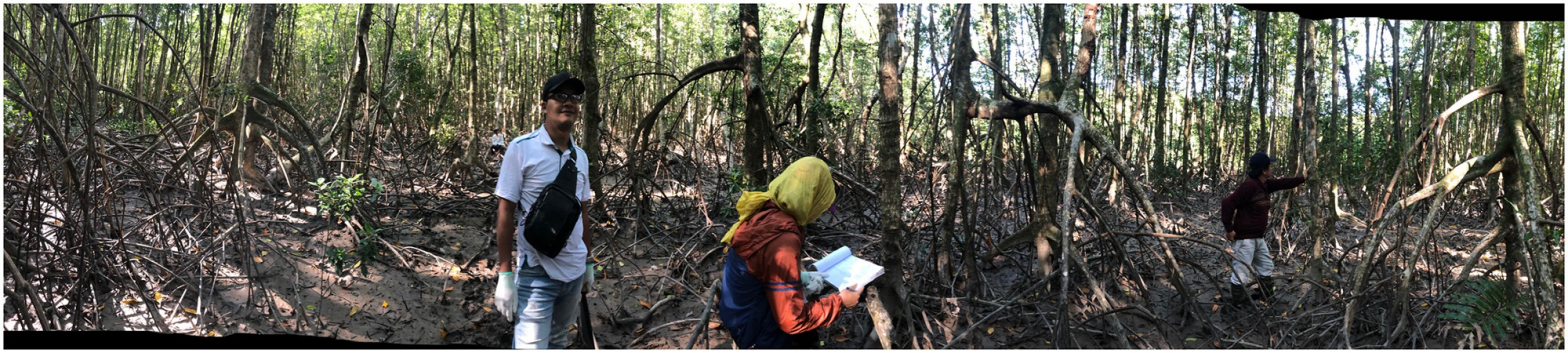
The survey in a sampling plot in the study area (Source: Photo taken by Pham Minh Hai (the author) in the study area in 2019).

To enter Mui Ca Mau National Park to take the field survey (Fig 2), the field permit was obtained from the Mui Ca Mau National Park Management Department (a division of the People’s Committee of CaMau) Field measured data from 18 sample plots (31 x 31m) were collected to validate the accuracy of the classification results. The study followed regulations from Circular No. 33/2018/TT-BNNPTNT on Forest survey, inventory and forest transition issued in 2016 by Vietnam Ministry of Agriculture and Rural Development (MARD) for the sampling plots. The sampling plots characterized by the average height of tree 15–25m, and distributed with high density included: *R*. *apiculata*, *A*. *alba*, mixed *R*. *apiculata—A*. *alba*, and mixed *A*. *alba—R*. *apiculata*. Ground truth surveys were conducted using Global Positioning System (GPS) to mark the position of tree in the plot sites.

## Methods

### Satellite image classification

**Fig 3:**
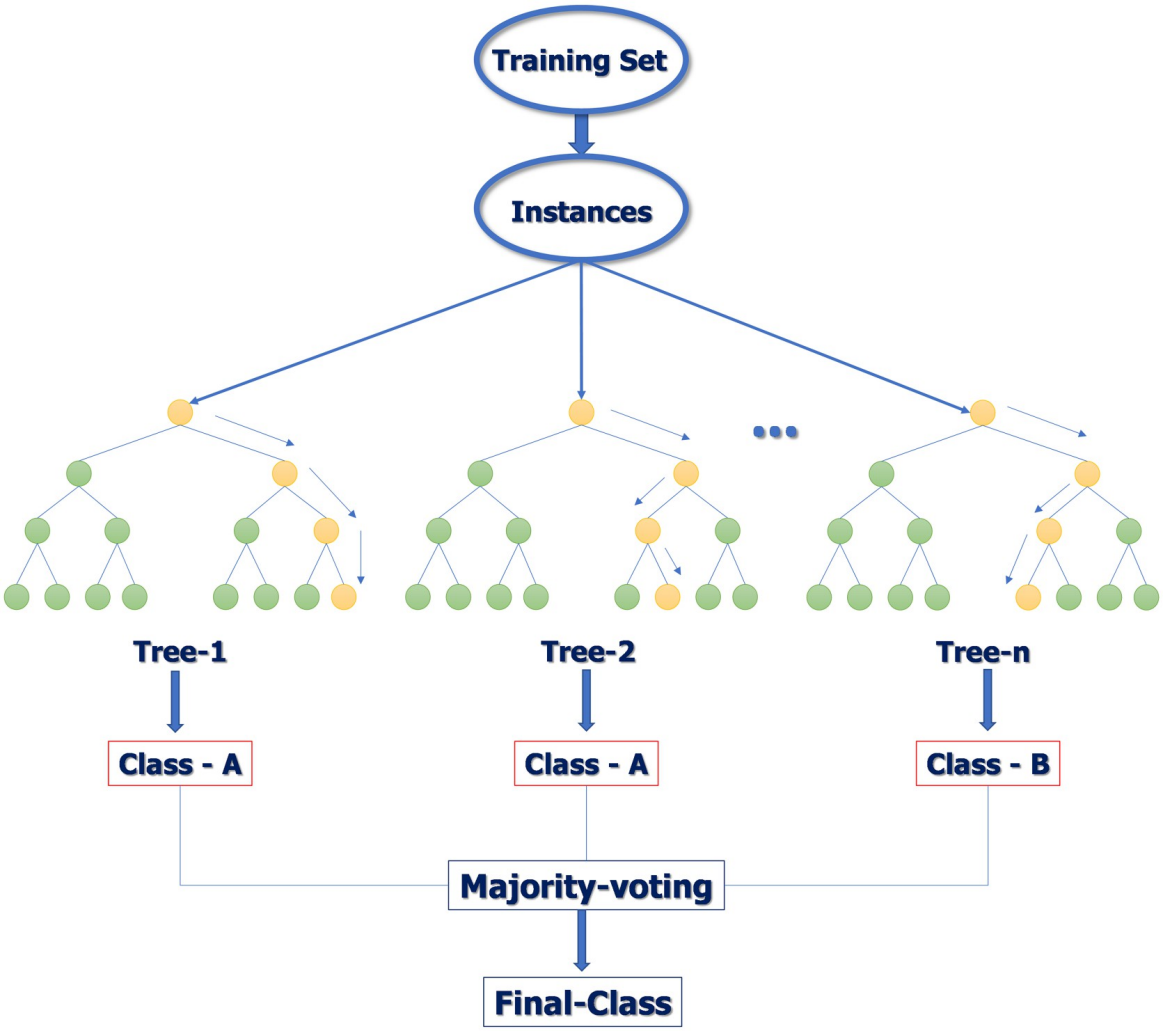
Flow chart of classification using Random Forest algorithm (Source: https://www.section.io/engineering-education/introduction-to-random-forest-in-machine-learning/).

Nowadays, employing machine learning algorithms with training datasets with a higher spatial resolution to classify mangrove forest is an accurate and low-cost way [12]. Random Forest is a supervised method, a combination of a number of non-parametric classification and decision tree/CART (classification and regression trees). This study selected Random Forest to classify satellite images because it can provide a higher quality of classification than linear classifiers and has been employed previously to map and classify mangroves [6, 34, 35]. Random Forest grows decision trees for classification. To classify a new object, the training sample is run through each decision tree in the forest. Each tree gives a classification, and the forest chooses the classification having the most votes. Also, Random Forest provides a robust algorithm for classifying large datasets. Decision tree is similar with hierarchy, composed of root node, including all samples, node separator which has decision rules, and the end of the leaf node, which represents desired classes (Fig 3).

In Ca Mau, there are four main types of mangroves: *R*. *apiculata*, *A*. *alba*, mixed *R*. *apiculata*—*A*. *alba* and mixed *A*. *alba—R*. *apiculata*. Most of mangrove forest was restored from the last 30 years.

**Fig 4:**
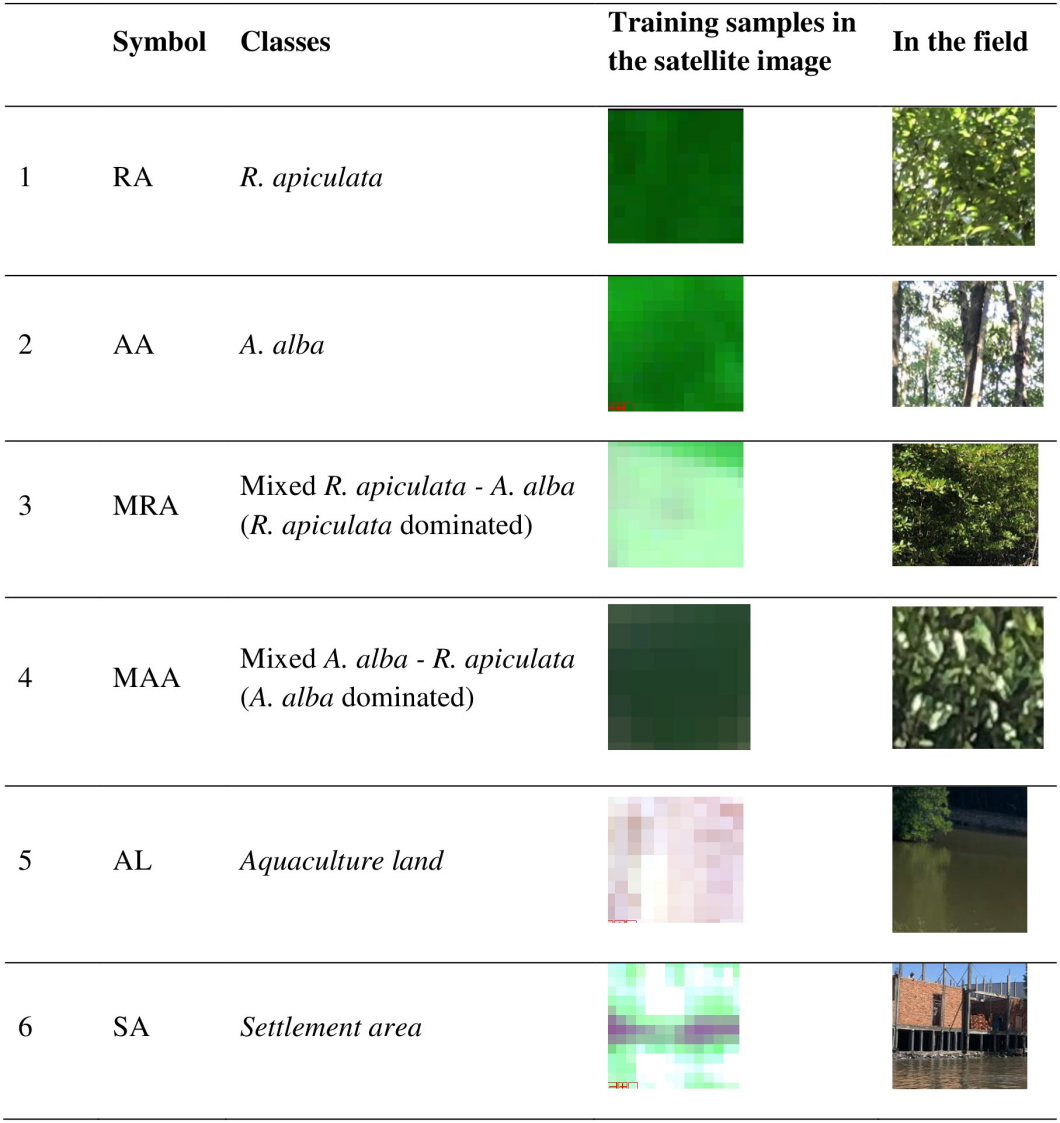
The illustrations of some training samples for the satellite image classification.

**Fig 5:**
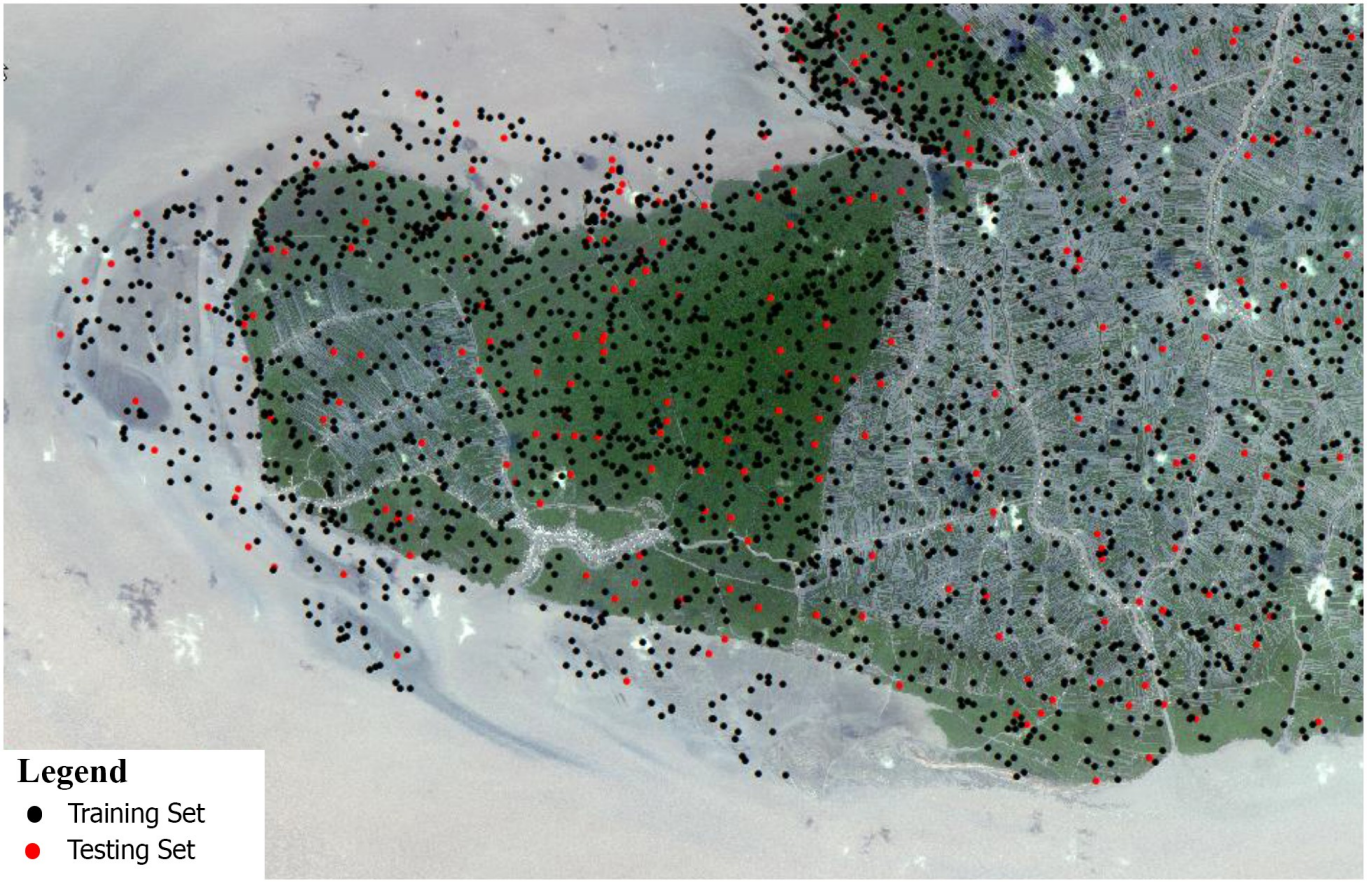
Sample collection for the random forest algorithm input (republished satellite data under a CC BY license, with permission from the Department of Remote Sensing, Ministry of Natural Resources and Environment of Vietnam, original copyright [1995, 2004, 2015]).

A field survey was conducted to collect samples for the image classification with the classes shown in Fig 4. A total of 300 sites were samples, in which 70% for training and 30% for accuracy validation (Fig 5). We used 2 methods to assess accuracy of classification results: 1) The visual comparison with Mangrove Statistic Map 2015 provided by Forest Inventory and Planning Institute (FIPI), a division of MARD and 2) The use of the confusion matrices by measuring, producer, user, and overall accuracy.

### Determining factors influencing mangrove health

**Fig 6:**
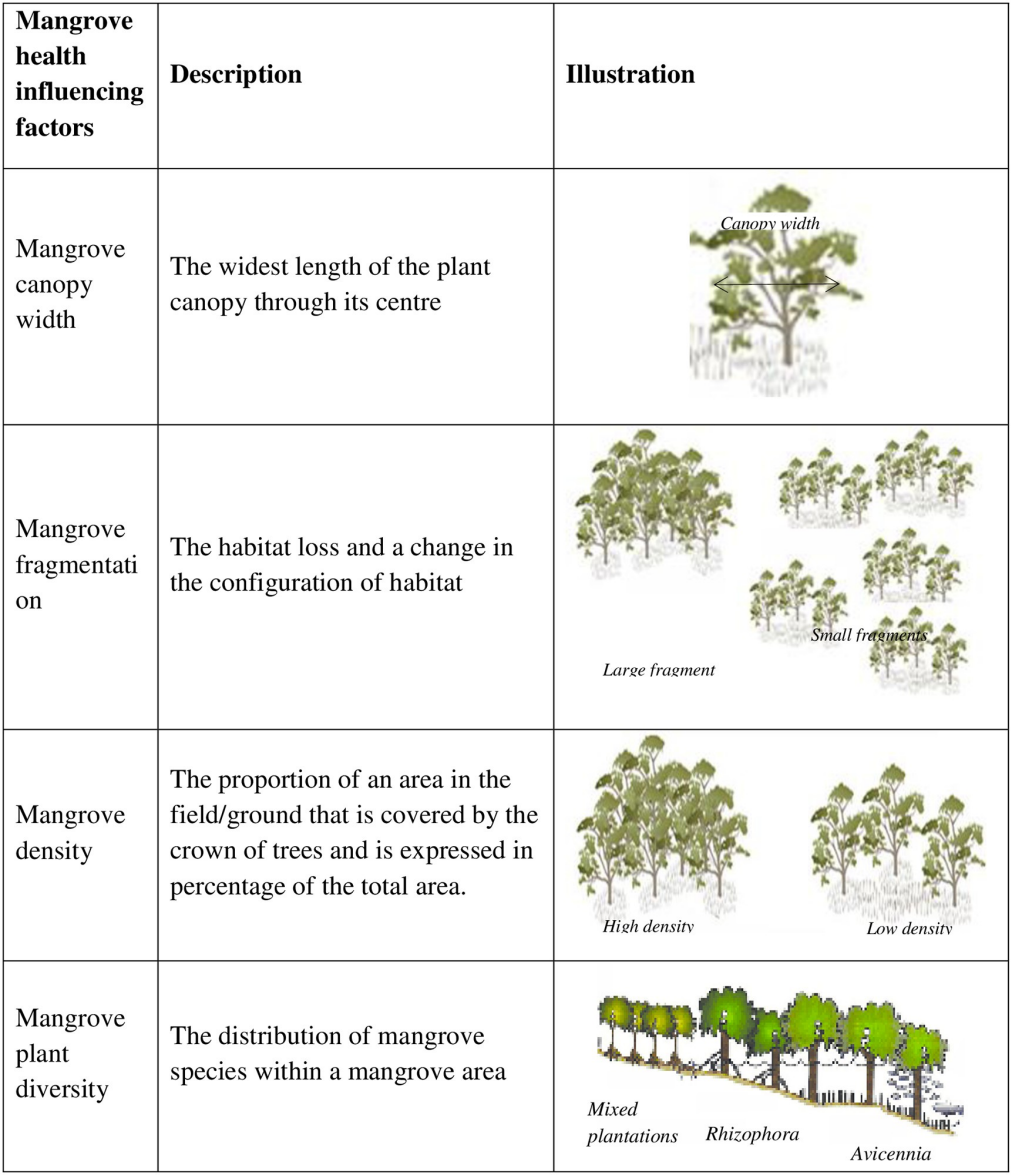
Indicators for the mangrove health assessment.

In Mui Ca Mau, mangrove health status affected by complex interactions of both natural and artificial factors, especially local people activities included reclamation, charcoal production, timber production, the conversion to shrimp farms. The present study reviews four factors such as mangrove canopy width, mangrove fragmentation, mangrove density [36], mangrove plant diversity [37] from peer-review scientific articles (Fig 6).

#### Spatial metrics and the mangrove health map

In this study, we used spatial metrics developed by McGarigal et al. (2002) [26] to evaluate the mangrove forest composition parameters, because they employed a series of quantitative indices representing physical characteristics of the landscape mosaic. Previous studies demonstrated that the relation between the spatial distribution of the forest patch and its change process can be visualized [24]. Spatial metrics can be computed as patch-based indices belonged to 6 major groups as: Area Metrics; Edge Metrics, Shape Metrics; Core Area Metrics; Contrast Metrics; Diversity Metrics; Aggregation Metrics [26].

**Fig 7:**
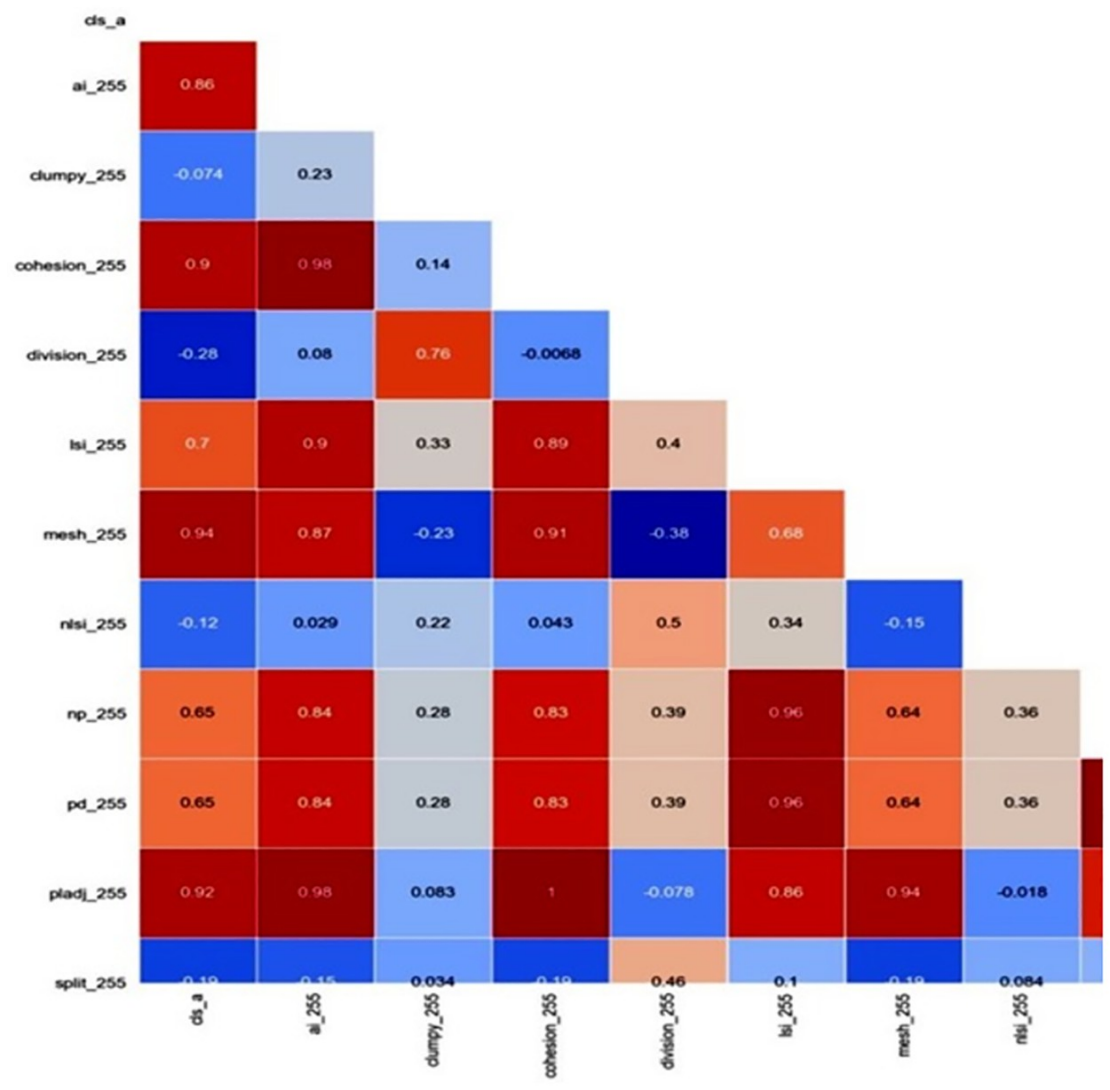
The Percentage of Like Adjacency index (PLADJ) showed high correlation coefficient in the Heat map.

In order to select suitable indices to assess mangrove health, we used a statistics solution called Pearson’s Correlation Coefficient [38]. The correlation coefficient has to be calculated for the pair-wise comparison included satellite image classification and maps derived from the spatial metrics [26]. We built a program to calculate the correlation coefficient and generate a Heatmap showing the degree and direction of the correlation (Fig 7) by using Python with libraries such as Rasterio, Seaborn, Numpy, Pandas, and Sklearn machine learning libraries. All the linear regression models between the spatial metrics and distribution of mangrove (CLS) fitted the data well (R^2^ > 0.9).

The digital classification results were used to calculate and analyze the spatial metrics of mangrove patches (Table 1). A 5×5-pixel-moving window was used to compute the value of mangrove characterizations; last value was returned to the center cell of the window. Indices pixel moved randomly conditional probabilities through the pixels in the moving window, with each calculation involving like adjacencies between four pixels; orthogonal cells were counted, but diagonal cells were ignored [28].

**Table 1:** Description of the spatial metrics used in this study (McGarigal et al. 2002).

Factors	Spatial metrics	Units	Range
Mangrove canopy width	Contiguity Index (CONTIG)	$\text{CONTIG}=\frac{\left[\frac{\sum_{\text{r}=1}^{\text{z}}{\text{c}}_{\text{ijr}}}{{\text{a}}_{\text{ij}}}\right]-1}{\text{v}-1}$	0 ≦ CONTIG ≦ 1
		c_ijr_ = contiguity value for pixel r in patch ij.	
		v = sum of the values in a 3-by-3 cell template.	
		a_ij_ = area of patch ij in terms of number of cells.	
Mangrove fragmentation	Percentage of Like Adjacencies (PLADJ)	$\text{PLADJ}=\left(\frac{{\text{g}}_{\text{ii}}}{\sum_{\text{k}=1}^{\text{m}}\;{\text{g}}_{\text{ik}}}\right)\left(100\right)$	0 ≦ PLADJ ≦ 100
		g_ii_ = number of like adjacencies (joins) between pixels of patch type (class) i based on the double-count method.	
		g_ik_ = number of adjacencies (joins) between pixels of patch types (classes) i and k based on the double-count method.	
Mangrove density	Patch density (PD)	$\text{PD}=\frac{{\text{n}}_{\text{i}}}{\text{A}}(10,000)$	PD > 0
		N = total number of patches in the landscape.	
		A = total landscape area (m2).	
Mangrove plant diversity	Shannon’s Evenness Index (SHEI)	$\text{SHEI}=\;\frac{-\sum_{\text{i}=1}^{\text{m}}\left({\text{P}}_{\text{i}}\;{\text{lnP}}_{\text{i}}\right)}{\text{lnm}}$	0 ≦SHEI ≦ 1
		P_i_ = proportion of the landscape occupied by patch type (class) i.	
		m = number of patch types (classes) present in the landscape, excluding the landscape border if present.	

#### Mapping mangrove health by using the Analytic Hierarchy Process (AHP)

Evaluation of the stability of wetland ecosystems involves a series of evaluation indices, some of which can be quantitatively described and some of which cannot be measured directly. The best method to evaluate problems that involve a number of uncertain indices is the Analytic Hierarchy Process (AHP) [39], which is a decision-making tool designed to deal with complex, unstructured and multi-factor problems. The AHP involves ranking a set of indices with respect to an overall goal, which is broken down into a set of criteria and indices. The AHP procedure involves three basic steps: (i) design of the decision hierarchy, (ii) pairwise comparison of elements of the hierarchical structure, and (iii) construction of an overall priority rating [40].

### The calculation of the relative weights for mangrove influencing factors

To determine the relative weights, the study used a 1–4 preference scale [26]. Each comparison was then transformed to a numerical value. For example, if the criteria for shape constraints of mangrove were judged to be essential or strong importance then the value criteria with respect to very good quality of the mangrove was given a score of 4. According to Saaty’s method, the normalized weight vector w = (W_1_, W_1_,…, W_1_) is obtained by the following formula:

$${\bar{W}}_{i}=\;\sqrt[{n}]{M_{i}}\;\left(i=1,2,\ldots ,n\right)$$ (1)

Vector $\bar{W}={\left[{\bar{W}}_{1},{\bar{W}}_{2},\ldots ,{\bar{W}}_{n}\right]}^{T}$ normalization

$$W_{i}={\bar{W}}_{i}/\overset{n}{\underset{i=1}{\sum }}\;{\bar{W}}_{i}\;\left(i=1,2,\ldots ,n\right)$$ (2)

Where *W* = [*W*_1_, *W*_2_, …, *W*_*n*_]^*T*^ is the characteristic vector of our calculation.

### Assigning value of the mangrove health index (MHI)

In the stability evaluation of the wetland ecosystem using the AHP method, determination of the weight and index variables was conducted on the basis of the evaluation work. Because each index data from different sources had different dimensions and numbers of levels, it was very difficult to analyze and contrast the original data based on their quantitative values. In this study, we employed local experts to determine the score for the evaluation of an index for assessing mangrove health called mangrove health index (MHI). The index was ranked from 1 to 4, corresponding to 1 (worst), 2 (moderate), 3 (good), 4 (very good), respectively, which gave the core vector V = (4, 3, 2, 1). Here, very good was defined as mangrove forest being stable, high coverage, density, diversity, but low fragment. Vice versa, worst was defined as mangrove forest being not very stable, scattered distribution and high fragments. For the evaluated weight for 4 indicators, there was a corresponding membership degree vector, R_i_ = (r_i1_,r_i2_,r_i3_,r_i4_) [40, 41]

$${\text{W}}_{\text{i}}=\sum_{\text{j}=1}^{4}\;{\text{R}}_{\text{ij}}*{\text{V}}_{\text{i}}$$ (3)

The score vector W = (W_1_, W_2_, W_3_, W_4_) can be used to describe each weight of each indicator (Table 2).

**Table 2:** Classes of indicator value.

	Factors	Weight (W_i_)	Chracteristics of mangrove forest	Intensity of importance (Mi)	Ranks
1	Mangrove canopy width	0.3	The average crown diameter of plants in the rich mangrove rehabilitation forest or *perennial mangrove forest*: *> 8 m*	4	Very good
			The average crown diameter of plants in the rich mangrove rehabilitation forest or *perennial mangrove forest*: *5–8 m*	3	Good
			The average crown diameter of plants in the rich mangrove rehabilitation forest or *perennial mangrove forest*: *3–5 m*	2	Moderate
			The average crown diameter of plants in the rich mangrove rehabilitation forest or *perennial mangrove forest less than*: *3 m*	1	Worst
2	Mangrove fragmentation	0.4	Fragmentation of patches for the rich mangrove rehabilitation forest or *perennial mangrove forest*: 80–100%	4	Very good
			Fragmentation of patches in the rich mangrove rehabilitation forest or *perennial mangrove forest*: 65–79%	3	Good
			Fragmentation of patches in the rich mangrove rehabilitation forest or *perennial mangrove forest*: 51–78%	2	Moderate
			Fragmentation of patches in the rich mangrove rehabilitation forest or *perennial mangrove forest*: *less than* 50%	1	Worst
3	Mangrove density	0.2	> 800 trees	4	Very good
			600–800 trees	3	Good
			200–600 trees	2	Moderate
			< 200 trees	1	Worst
4	Mangrove plant diversity	0.1	Mixed forest with more than 2 mangrove species	4	Very good
			Mixed forest with 2 mangrove species	3	Good
			Forest with 1 mangrove species	2	Moderate
			Bare land	1	Worst

To obtain the evaluation result, it is necessary to choose the appropriate method for the assessment. The current assessment methods used 4 factors (Table 2) with its corresponding weight. The mangrove health index (MHI) calculation is:

$$\text{MHI}=\sum_{1}^{4}{\text{W}}_{\text{i}}*{\text{V}}_{\text{j},\text{k}}$$ (4)

Where:

MHI = Value in the mangrove health map;

W_i_ = Weight of factors;

V_j,k_ = Pixel value in each factor map;

Fig 8.

**Fig 8:**
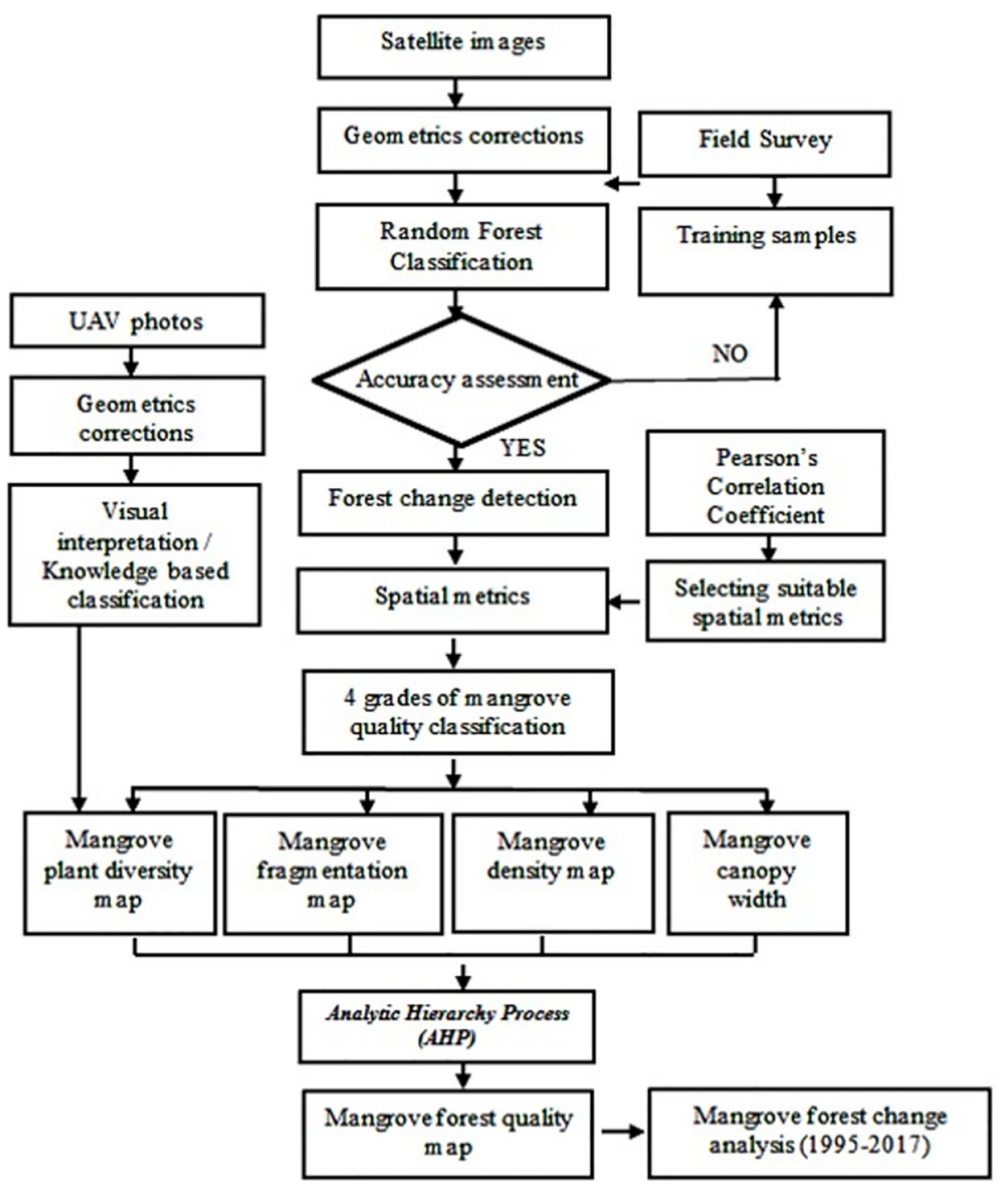
The flow chart developed in the study.

## Results

### Land use classification

**Fig 9:**
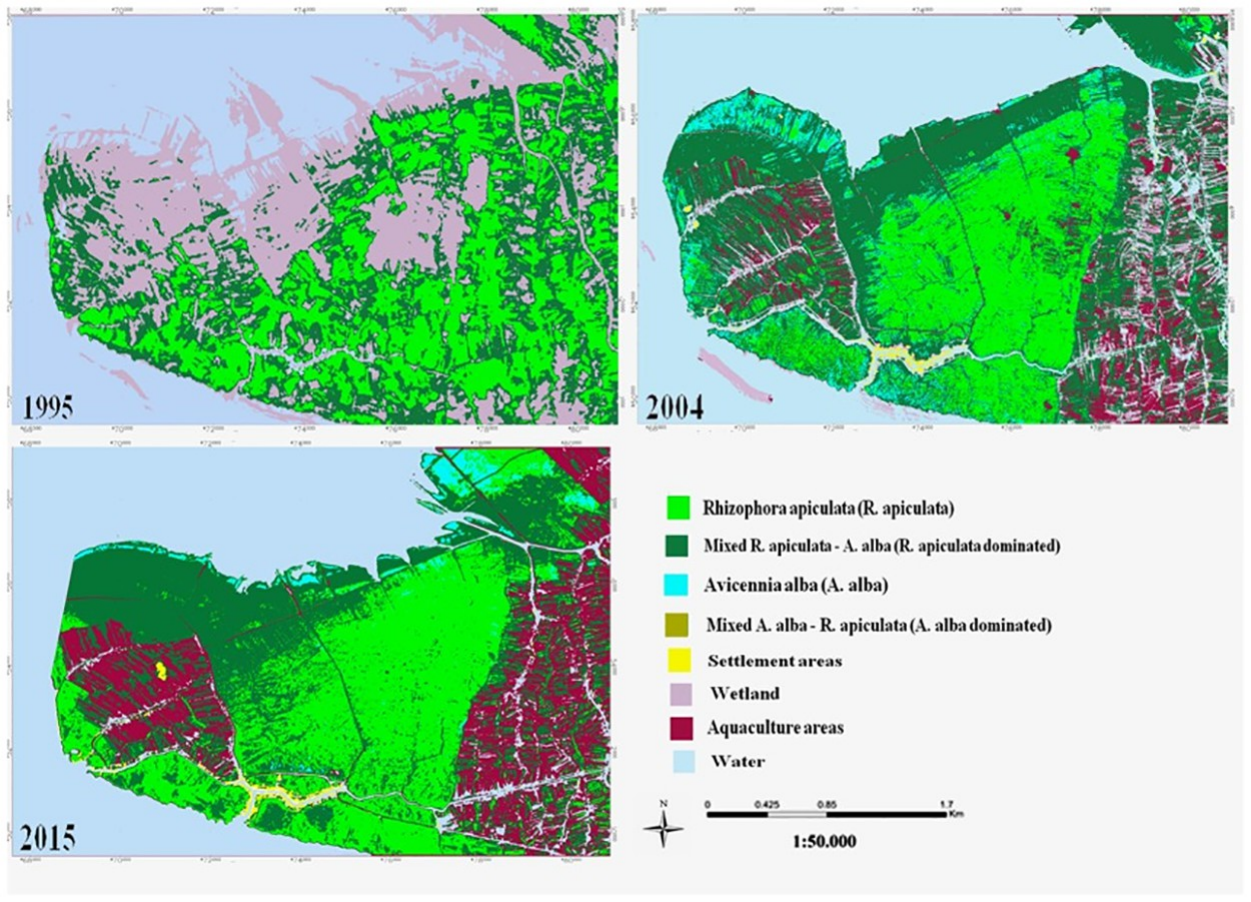
Satellite image classification results (republished satellite data under a CC BY license, with permission from the Department of Remote Sensing, Ministry of Natural Resources and Environment of Vietnam, original copyright [1995, 2004, 2015]).

**Fig 10:**
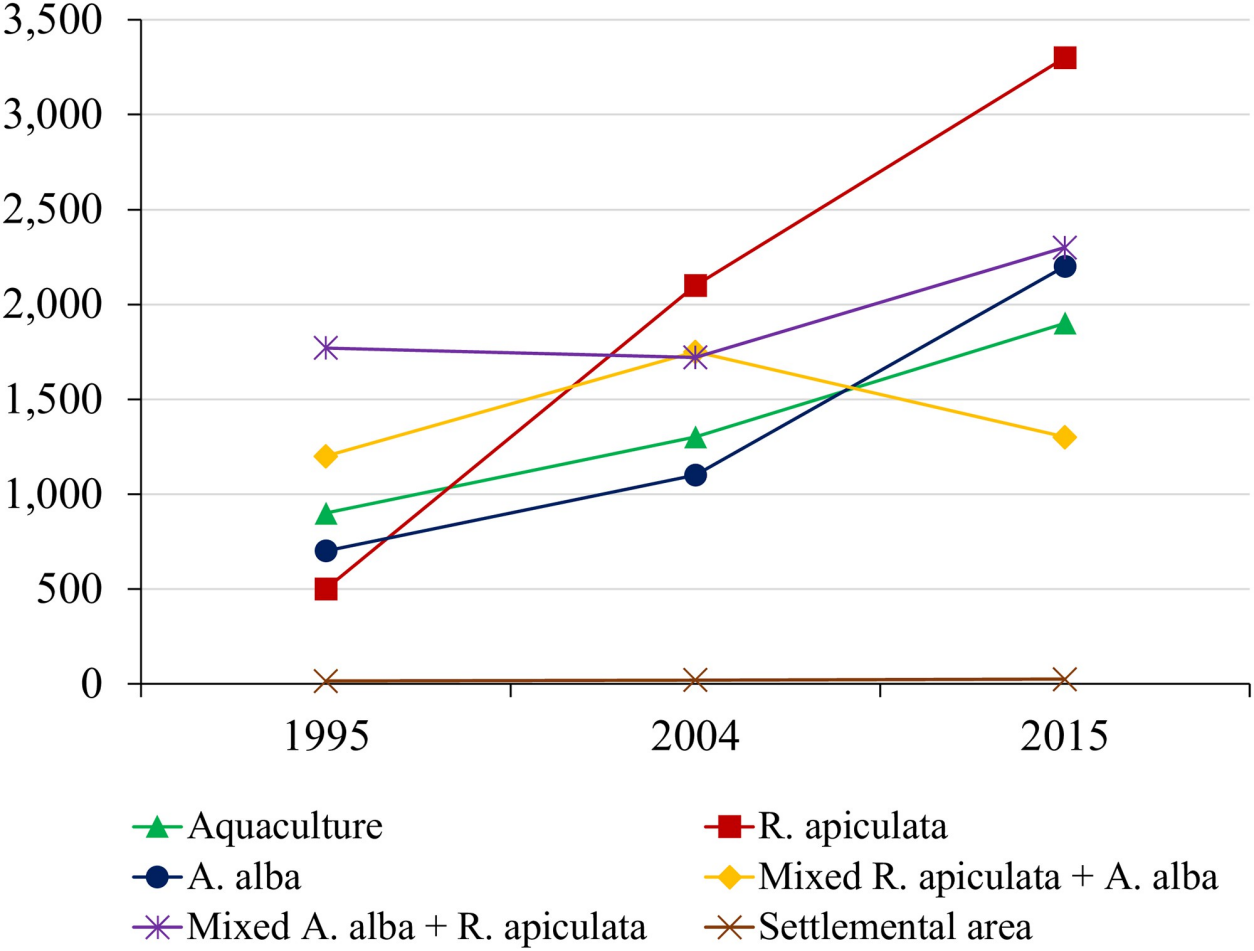
Change of mangrove areas in Ca Mau from 1995 to 2015.

On the classification methods described above, random forest (RF) (described in the part 3.1) was used to map mangrove land use for the satellite image in 1995, 2004, and 2015. The classification results and fluctuations of land use classes overtime shown in Figs 9 and 10, respectively.

**Fig 11:**
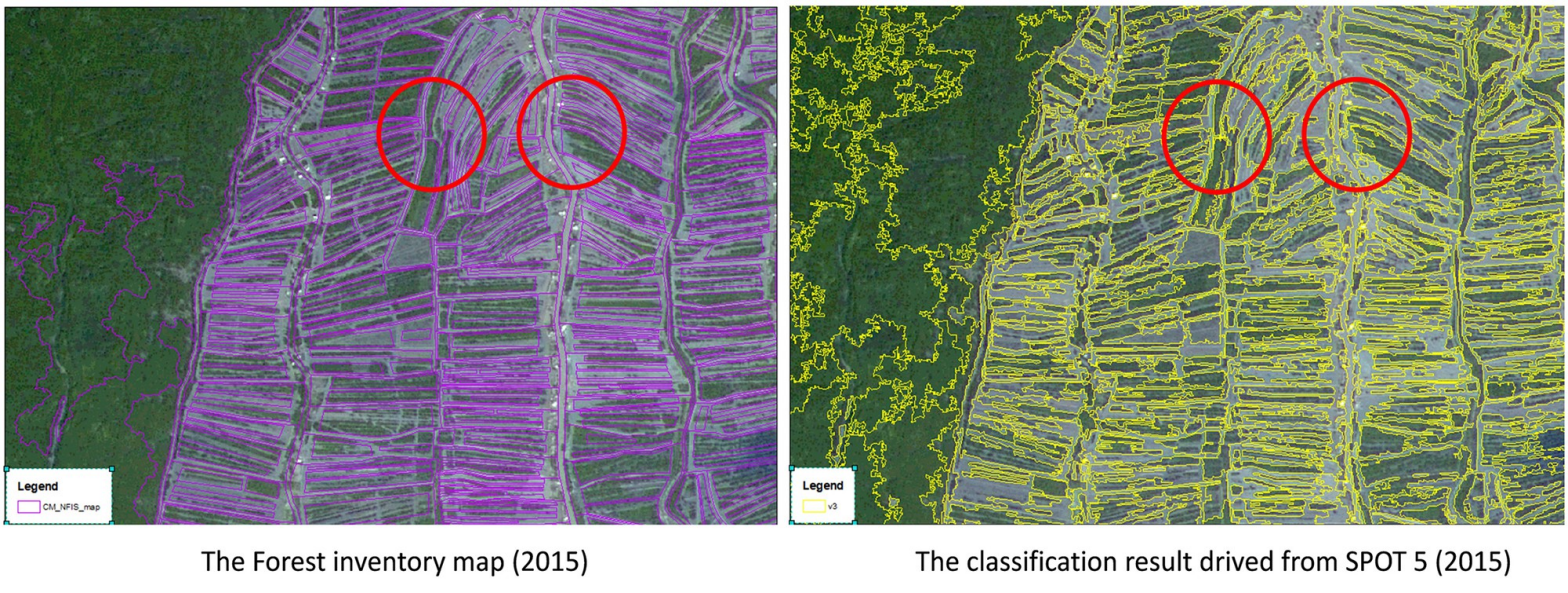
The visual comparison between the results of the image classification and the Forest inventory maps (republished satellite data under a CC BY license, with permission from the Department of Remote Sensing, Ministry of Natural Resources and Environment of Vietnam, original copyright [1995, 2004, 2015]).

The forest inventory maps of the year 1995, 2005, 2010, 2015 provided by Forest Inventory and Planning Institute (FIPI) were used to validate accuracy of the classification results by comparing mangrove boundaries among classification and FIPI maps. The results show that the classification results are more accurate with a high level of detail about the boundaries of the mangrove area (Fig 11). In contrast, many areas with mangrove boundaries on the FIPI map of the same period were highly generalized at the time of establishment, thus not accurately reflecting the mangrove area boundaries (red circle in Fig 11).

In addition to, the confusion matrices of the classifications were also investigated, to provide an in-depth statistical assessment in each class. The results of the assessment of accuracy when verifying with checking pixels are as follows: the producer accuracy (PA), the user accuracy (UA), and the overall accuracy (OA) (Table 3).


$$\text{OA}=\left(47+18+34+13+28+29\right)/222=80\%$$ (5)

**Table 3:** The confusion matrices of classified classes.

No.	Legend	Classes	Rnmd	Rnmm	Rnmhgdm	Rnmhgmd	Ntts	Dt	Total	PA	UA
1	*R*. *apiculata*	**Rnmd**	47	7	3	0	0		57	82%	94%
2	*A*. *alba*	**Rnmm**	1	18	6	0	0		25	72%	64%
3	Mixed *R*. *apiculate—A*. *alba*	**Rnmhgdm**	2	3	34	4	1	4	48	71%	63%
4	Mixed *A*. *alba—R*. *apiculata*	**Rnmhgmd**	0	0	5	13	1	5	24	54%	59%
5	Aquaculture land	**Ntts**	0	0	1	1	28	0	30	93%	93%
6	Settlement area	**Dt**			5	4	0	29	38	76%	76%
Total			50	28	54	22	30	38	222		

### The mangrove forest health map

#### Mapping mangrove health in Mui Ca Mau, Ca Mau province of Vietnam

**Fig 12:**
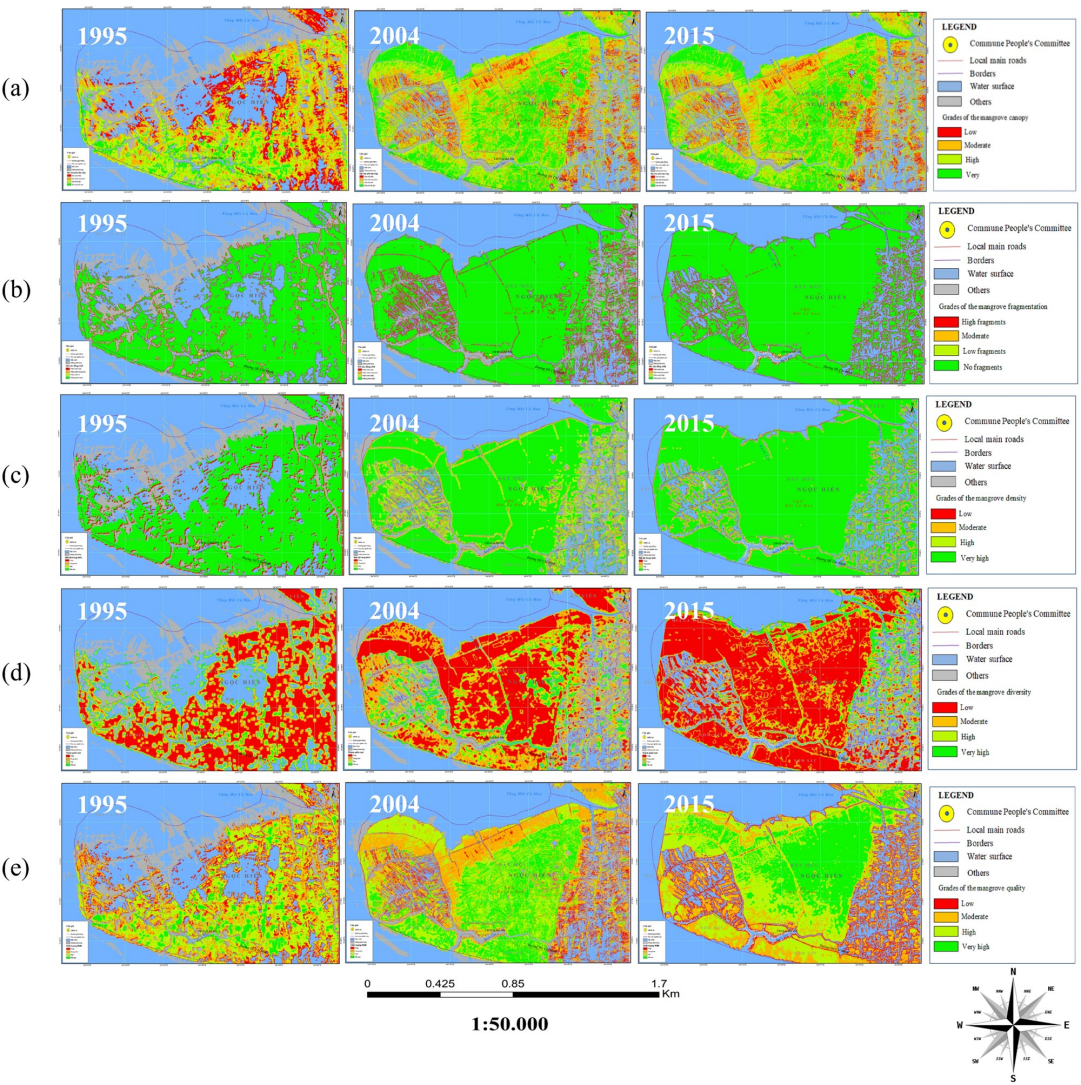
Mapping factors influencing mangrove health in Ca Mau: (a). The Mangrove canopy width map; (b). The Mangrove fragmentation map; (c). The Mangrove density map; (d) The Mangrove diversity map; (e). and The Mangrove health map. (Republished satellite data under a CC BY license, with permission from the Department of Remote Sensing, Ministry of Natural Resources and Environment of Vietnam, original copyright [1995, 2004, 2015]).

To generate mangrove health map, the study used the Analytic Hierarchy Process (AHP) [39] with the calculation described in part 3.4. Fig 12e shows the mangrove health map of Mui Ca Mau, Ca Mau province of Vietnam.

## Discussion

### Characterizing the mangrove health change in Mui Ca Mau from 1995 to 2015

In the study, mangrove health refers to the composition of a mangrove community in terms of mangrove canopy, fragmentation, density, and species. From Fig 12, we can see changes of each mangrove health influencing factor corresponding to its level of quality overtime. The change of each mangrove health influencing factor in each period led to forest vigour or vice versa. The period from 1995 to 2004 witnessed a positive change in mangrove health from low to moderated level (Fig 12e). We can see an increase in the mangrove canopy (Fig 14a) from low to high level reflected by a threefold increase of mangrove areas from 4121 ha to 6640 ha. *R*. *apiculata* and *A*. *alba* had significantly changed from 535 ha to 2100 ha and 687 ha to 1100 ha, respectively (Fig 10). This trend can be explained as the west of Ca Mau (border to the Gulf of Thailand) had very high accretion rate, about 32.2 m/year. The land strip was widened by nearly 6 km in width and 7 km in length during this time period [32]. The total accretion area was 8318 ha, of which 1466 ha are planted by *A*. *Alba* [42].

**Fig 13:**
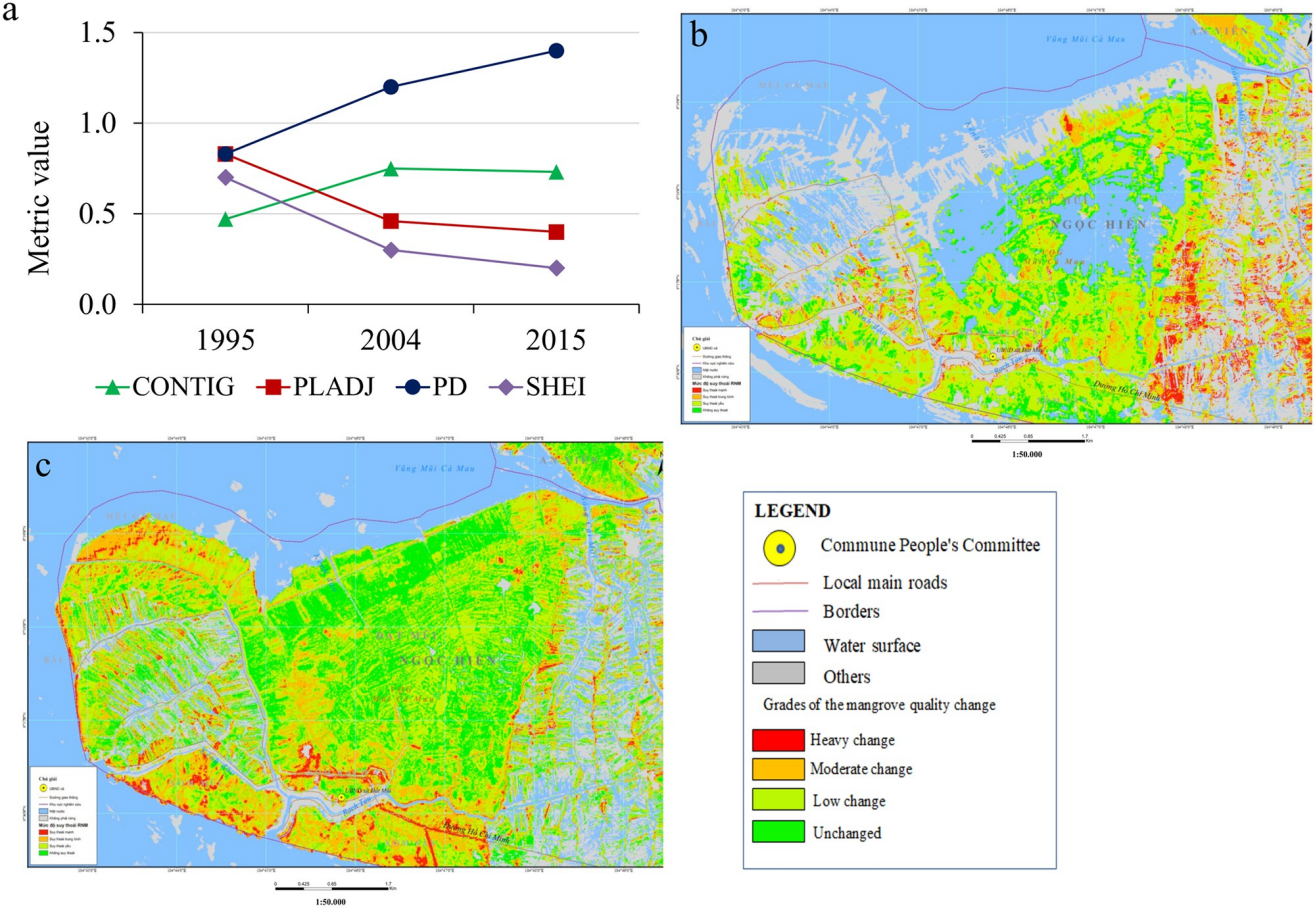
(a). Changes of spatial metric indices from 1995 to 2015, (b). Mangrove health change detection maps from 1995 to 2004, (c). Mangrove quality change detection maps from 2004 to 2015. (Republished satellite data under a CC BY license, with permission from the Department of Remote Sensing, Ministry of Natural Resources and Environment of Vietnam, original copyright [1995, 2004, 2015]).

**Fig 14:**
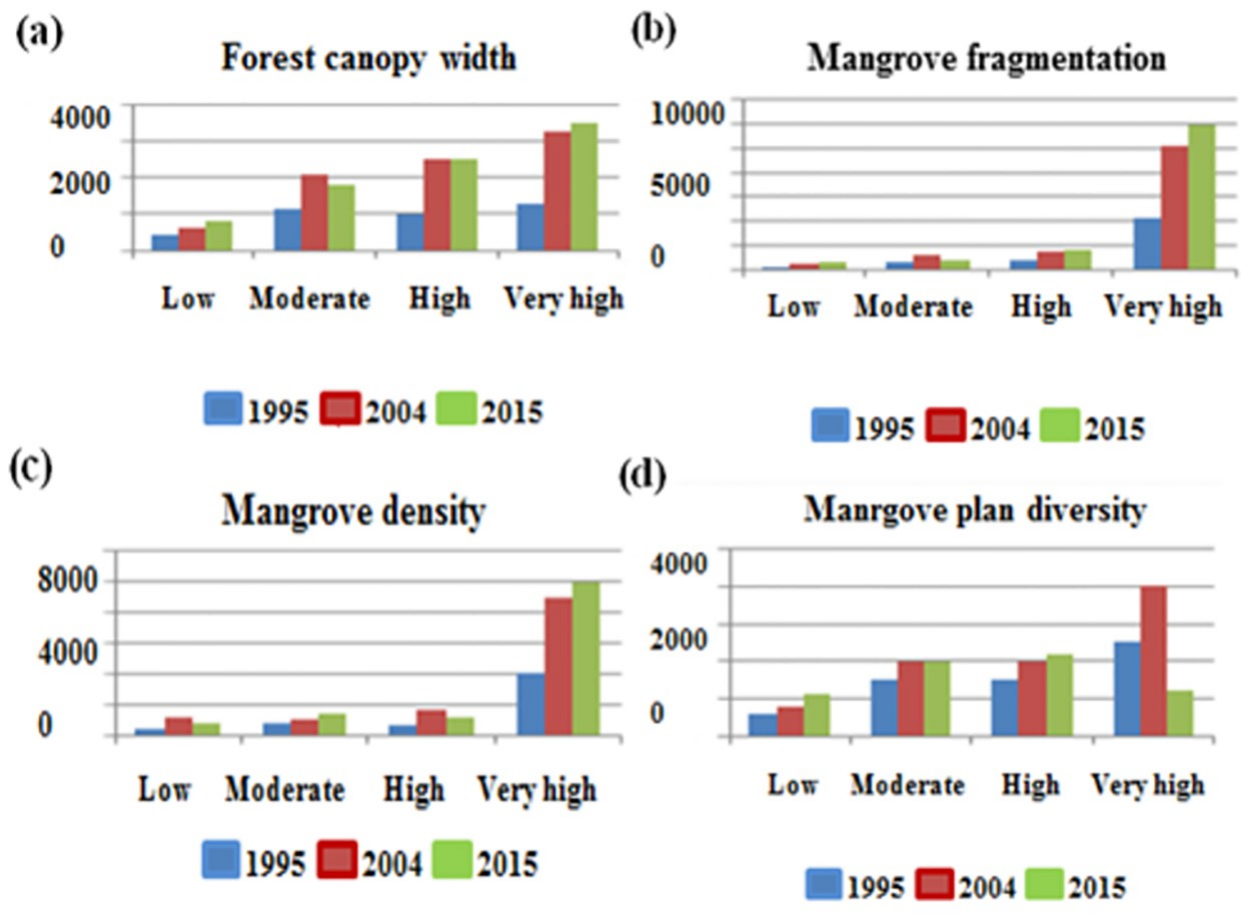
Changes of the mangrove health influencing factors overtime.

In contrast to a dramatic increase in mangrove areas, distribution of mangrove in 1995 was heterogeneous and relatively undeveloped, as indicated by the low values obtained from mangrove fragmentation (Fig 14b). By 2004, mangrove health was improved, and illustrated by a moderated change (Fig 12b). The PD increased in concern with decrease in PLADJ (Fig 13a) indicating that the forest started to diffuse outward to become mixed *A*. *alba—R*. *apiculata* forest in the north (Fig 9). The forest areas became more homogeneous with high density (Fig 14c), although there was a development of forest patches some distance away to the north. Also, this period experienced a slightly change to expand aquaculture land further to the north (Fig 9), as a result there was a significant change from the conversion of mixed *A*. *alba—R*. *apiculata* forest to shrimp farms. Increase of aquaculture land from 920 ha to 1340 ha in 1995 and 2004 highlighted to this trend (Fig 10).

The characteristics of mangrove heath status in Mui Ca Mau from 2004 to 2015 showed a good condition in both quantity and quality (Fig 12e). During this period, the mangrove canopy changed from moderated to very high level. The forest areas increased from 6060 ha to 8604 ha. Although Mui Ca Mau lost 1295 ha of mangrove, but 3259 ha (2.5 times the forest lost) was reforested by mangrove restoration projects. By 2015, *R*. *apiculata* and *A*. *alba* were the dominant species in Mui Ca Mau, the lowest value of SHEI supported this opinion (Fig 12a). Both of them had a marked increase from 2100 to 3260 ha (64%) and 1100 to 2169 ha (50%) (Fig 10). *R*. *apiculata* mostly distributed in the center of Ca Mau National Park, while *A*. *alba* distributed near the east and west coasts of Ca Mau, which led to the development of *A*. *alba* forest in the west coast, and mixed *A*. *alba—R*. *apiculata* forest in the east coast of the study area (Fig 9).

**Fig 15:**
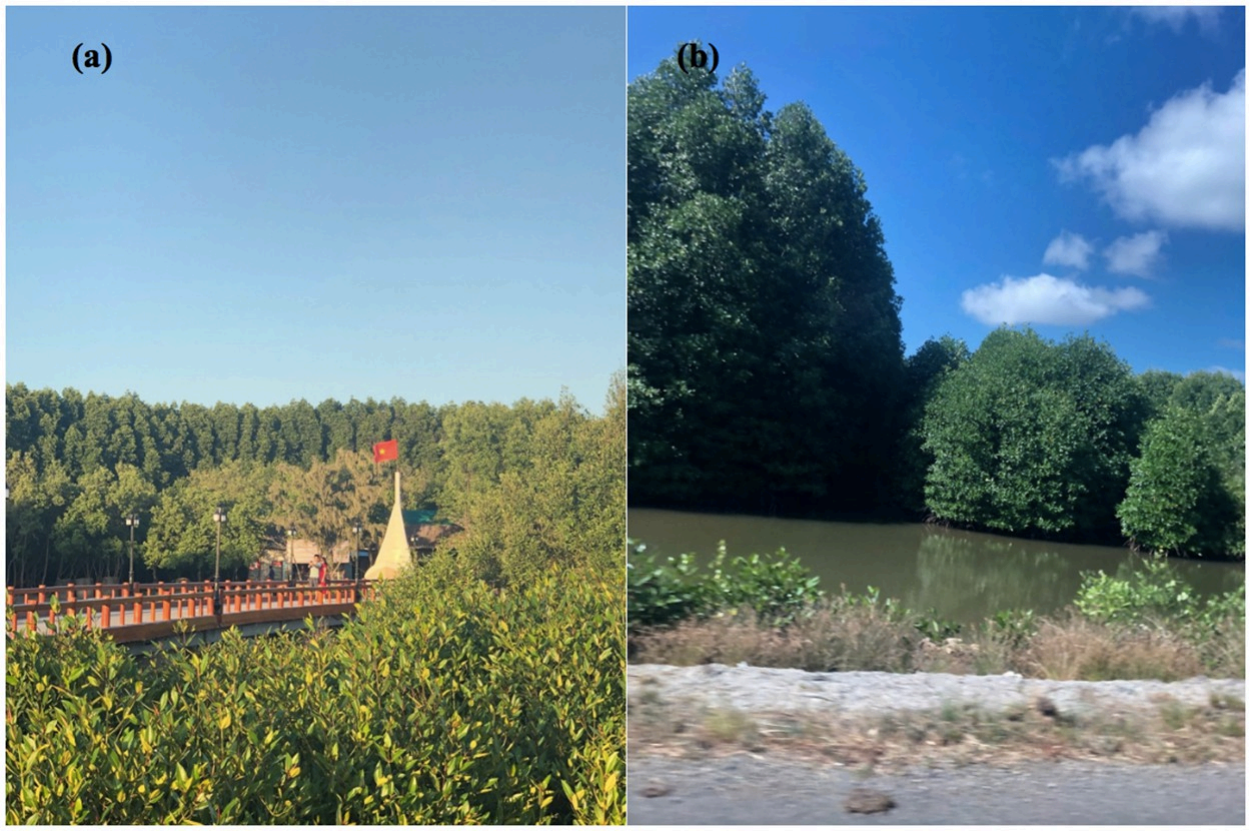
The bridge to the landmark at the end of the National 1^st^ highway (left side) and ecological forest shrimp farms (right side).

To highlight to this trend, we can analyst a transition from *A*. *alba* forest to *R*. *apiculata* forest and mixed *A*. *alba—R*. *apiculata* forest (Fig 9). From 2004, *A*. *alba* was planted and grew rapidly in the west coast of Ca Mau. Then, *R*. *apiculata* tended to develop outward from the middle to the west coast, and gradually invaded *A alba* to become to *R apiculata* forest by 2015. On the contrast, the *R*. *apiculata* forest in the east coast gradually encroached by *A*. *alba* into mixed *A*. *alba—R*. *apiculata* forest (Fig 9). A decline in PLADJ and an increase in PD and PD from 48% to 40% and 1.3 to 1.8 (Fig 13a) indicated the mangrove areas more homogeneous and in better condition by 2015. However, Fig 14d indicates that degradation of mangrove species in some areas near Dat Mui town in the South of Ca Mau, where located a large area of mixed *A*. *alba—R*. *apiculata* forest. The impacts of local socio-development activities on the mangrove health were observed (Fig 15). The area dedicated to the end of national highway No.1, and the construction of new roads along were a driving force for the forest lost and degradation. The construction of national highway No. 1 created a wall that changed the hydrological regime, thus negatively affected the growth of mangroves.

### Implication for the mangrove management

In order to effectively protect and develop mangrove forests in Mui Ca Mau, it is necessary to review and develop forestry mechanisms and policies with clear objectives and orientations to encourage the formation of a forest management system with the participation of the State and local community. From the beginning of 1990, to slow the rate of deforestation across the country, the government announced Decision 286/QD-TTG in 1991 including urgent measures to protect and develop forest areas and plant new 5 million hectares of new forest across the country. However, the Decree 773-TTg announced in 1994 encouraged coastal areas for developing aquaculture. As a result, large area of mangrove forest in Ca Mau was destroyed. This policy encouraged shrimp farmers to clear more mangroves for aquaculture activities. The conflict between state policies led to the rapid expansion of shrimp farming in Vietnam in 2000s, especially in Ca Mau, where 202,000 hectares were used for shrimp farming [32, 43].

This study indicates that land use change in Ca Mau was mainly due to conversion from forest to shrimp farms, settlements areas and public constructions. Shrimp farms increased 41% from 920 ha in 1995 to 1900 ha in 2015 (Fig 10), resulting the forest pattern had become more fragmented (Fig 12b). The result of the MAM project created a driving force impacting to the mangrove forest by promoting ecological shrimp farming (Fig 15b) under mangrove forest with the combination of 70% of mangrove area with 30% of ecological shrimp farming area is a new method in sustainable aquaculture development. The mangrove-shrimp farming model has become the preferred model in Ca Mau, helping to promote forestry as well as the desire to promote the aquaculture industry.

Moreover, to ensure the sustainable development of shrimp farming, it was necessary to replant mangroves in shrimp farms. The restoration of mangroves must be strictly calculated according to the ratio of shrimp farming areas for each type of forest [32], and the recommend mangrove species such as *R*. *apiculata* and *A*. *alba*, which shows very strong vitality against coastal erosion, salt water, flooding and strong wind in the local areas.

## Conclusion

This study provides an advanced approach to map mangrove health in Mui Ca Mau, Ca Mau province of Vietnam. Firstly, we successfully exploited the structure in the satellite images for determining mangrove health and proposed appropriated spatial indices included Contig Index, PLADJ Index, PD index, SHEI index to assess the mangrove influencing factors included the mangrove canopy width, mangrove fragmentation, mangrove density, mangrove plant diversity. All spatial metrics correlated well with the distribution of mangrove (R^2^ > 0.9). Secondly, this study revealed that mangrove connectivity and configuration have a strong correlation indicating the mangrove health through the influencing factors. Analytic Hierarchy Process (AHP) shows its potential in supporting to establish mangrove health map from multiple parameters. Despite an increase in total mangrove area, we found that mangroves had become more fragmented in the study area during the study period due to land use conversion from mangroves to shrimp farms, settlements areas and public constructions such as bridges and roads. Finally, this study proved that remote sensing and spatial metrics can be used to assess the mangrove health effectively. The research results will help the local government to reduce manpower, time and cost in monitoring the health status of mangrove in Ca Mau compared to the current practice of checking manually the timber stock volume data as an indicator of mangrove health.

## Supporting information

S1 File (DOCX)

S2 File (RAR)
