# Supplementary material for: Mangrove health assessment using spatial metrics and multi-temporal remote sensing data
Source: PLoS One. 2022 Dec 6;17(12):e0275928. doi: 10.1371/journal.pone.0275928 (PMC9725159; doi:10.1371/journal.pone.0275928)
Supplement: S1 File — (DOCX) [file pone.0275928.s001.docx]

**Publication date:**

August 16, 2022

**DOI:**

DOI 10.5281/zenodo.6997305

**Keyword(s):**

[**remote sensing**](https://zenodo.org/search?q=keywords%3A%22remote+sensing%22)

**Subject(s):**

[**Remote sensing**](https://zenodo.org/search?q=subject.term%3A%22Remote+sensing%22)

**Published in:**

PLOS ONE:.

**Related identifiers:**

Cited by

- [10.5281/zenodo.6997305](https://doi.org/10.5281/zenodo.6997305) (Journal article)

**License (for files):**

- [Creative Commons Attribution 4.0 International](https://creativecommons.org/licenses/by/4.0/legalcode)
